# Medical News and Miscellany

**Published:** 1892-12

**Authors:** 


### Medical News and Miscellany.

Dr. Jas. Kemp has removed from Walnut to Thorp Springs, Texas.

---

Removed.—Dr. L. H. Hardy has removed from Paige, Texas, to Throckmorton, Texas.

---

Dr. Bruce P. McVey has removed from Ella, Brazos county, to Mumford, Robertson county, where he will in future reside.

---

Thermometric Observation.—"Mamma," said little Johnny, "if I swallowed a thermometer would I die by degrees?—*Exchange.*

---

Wanted.—"Wanted, a gentleman to undertake the sale of a patent medicine; the advertiser guarantees it will be profitable to the undertaker."—*Exchange.*

---

Just as We Expected—"We regret to have to announce the death of Mr. Cumso; but we are not astonished to hear of the sad event, as deceased had been attended for some time by Dr. Borax."—*Exchange.*

---

**The Story of an Actress.**—Madame Adelaide Ristori, the famous tragedienne, has written, for the *Ladies Home Journal*, two important autobiographical papers, in which she will tell "How I Became an Actress" and describe "The Methods of My Art."

---

**A New Nedofik Sofa.**—(See page 1, this issue), cost at factory $65, is for sale at this office for $45 ; never been used ; curled maple finish, upholstered in brown morocco, and nickel ffnish ; complete sofa and operating table. Reason—no use for it, not in general practice.

---

**The World's Fair Exposition.**—Department of Liberal Arts. The Bureau of Hygenia and Sanitation. The JOURNAL is indebted to Mrs. Rosine Ryan, the Texas Representative on the Board of Lady managers, for a pamphlet bearing the above title. A copy may be had by addressing Mrs. Ryan, at Palmer House, Chicago.

---

**American Public Health Association** — Officers elect: President, Dr. S. H. Durgan, Boston; 1st Vice-President, Dr. Edw. Liceago, City of Mexico; 2nd Vice-President, Dr. Emanuel P. Lachapelle, Montreal, Can.; Secretary (re-elected), Dr. Irvin A. Watson, Concord, N. H.; Treasurer, Dr. Henry D. Holton, Brattleboro, N. Y.

---

**Married in New York**, Thursday, December 8th, inst., at 8:30 p, m., Dr. William Wayne Ashhurst to Ellen Eyre Gaillard, daughter of Mrs. Mary E. Gaillard, and the late Prof. E. S. Gaillard. The JOURNAL acknowledges the courtesy of an invitation, and extends its best wishes for the happiness and prosperity of the young couple.

---

**Recovering.**—The JOURNAL is gratified to learn that the son of its friend and patron Dr. E. S. Adams of Garrison, who met with such a serious railroad accident early in the fall, whereby his leg and foot were injured and stripped almost completely of integument, is making a better recovery than was hoped for; the skin-grafts having been successfully applied, the entire surface is now nearly re-covered.

**Texas Students**, by their close application and study, are well to the front in the various colleges always, and the JOURNAL has had occasion, more than once, to announce the award of prizes to some Texas boys. In the Medical Department of the University of Tennessee, we are advised, there is a full attendance of Texans this session, and Dr. Phil R. Simmons, of Sipe Springs, Texas, has been elected Valedictorian. The school is in a flourishing eondition.

---

**Richard Swearingen Robinson.**—Born to Mr. and Mrs. Eugene Bremond Robinson, on ——th October, '92, a son. Mrs. Robinson is the only daughter of Dr. R. M. Swearingen, the popular and gifted State Health Officers and Surgeon General of Texas. The happy young parents have bestowed his name upon their son. May he live long and prosper, and inheriting his noble traits of character, become as great and as good a man as his grandfather, is the JOURNAL'S sincere wish.

---

**Dr. J. B. Murfree**, of Murfreesboro, Tenn., Chairman Tennessee State Board of Medical Examiners, has resigned the position to accept a chair in the Medical Department of the University of the South at Sewanee. He is extensively known throughout the United States, and especially in the South, as a man of high culture and splendid professional ability, and is not unknown to fame in polite literature. When his daughter, under the *nom de ptume* of "Chas. Egbert Craddock," published her stories "In the Tennessee Mountains," many persons thought it was [the product of the Doctor's pen. Dr. Murfree is a valuable acquisition to the Faculty, upon which they and the school are to be congratulated.

---

**Death of Dr. Francis.**—Dr. C. C. Francis, of Cleburne, President of the Johnson County Medical Society, died at his residence in Cleburne on the — day of November, 1892, after a long illness. Dr. Francis was held in high esteem by all who knew him. He was a good physician, a good friend, a provident father and a useful member of society, and his death causes a vacancy hard to fill. The Johnson County Medical Society held a meeting and passed the most complimentary resolutions, testifying to the many virtues of their departed brother and the high esteem in which he was universally held. He was also an old and honored member of the Texas State Medical Association and one of the earliest and staunchest friends of the JOURNAL. The

JOURNAL extends its sincere sympathy to Dr. Francis' family and to the good people of Cleburne in their great loss.

---

**For Sale.**—An opportunity to have a healthy, quiet and comfortable home, with a regular practice from the start of $2000 a year in a section of Texas that has the combined resources of the grain of tae North, cotton of the South, and cattle of the West. Churches, school, Masonic lodge, cheap lands, a good opportunity to embark in the stock business. A bonanza for the wornout practitioner of the malarial districts who is seeking health, and wishes to continue in practice. Improved acre lot, dwelling four rooms, two porches, chimney, cistern, garden, orchard, out-houses, and well, desirably located. Opposition weak. Terms, $1000. Will remain till purchaser is thoroughly satisfied he can hold the field. Object, post graduate course. Address, W. B. ANDERSON, M. D.,
Content, Runnels county, Texas.

---

**Dr. A. E. Spohn.**—Attention is called to the advertisement of Dr. Spohn's Sanitarium in this issue. Dr. Spohn was educated at McGill University, Montreal, in the University of Michigan, Long Island College Hospital, and the Bellevue Hospital Medical College, N. Y. In 1867, he was a member of the Faculty of the Long Island Medical College and Hospital, filling the chair of Assistant Professor of Surgical Anatomy. He has devoted much time to surgical work, and visited Europe in 1888, where he attended the hospitals of Paris and other cities. In 1890, he spent the winter in Philadelphia, devoting himself principally to gynecological work. He opened a private hospital at Corpus Christi and met with such success—receiving patients from a distance—that he is induced to devote himself exclusively now to the one branch, Surgical Diseases of Women. Dr. Spohn performed Cæsarian section in a case of malacosteon which is the first operation of the kind ever performed or attempted in the United States, so says Dr. Robt. P. Harris.

---

**Distinguished Texas Physicians at the A. P. A. Meeting in Mexico.**—When three of the Austin delegation entered the convention hall—Drs. Bennett, McLaughlin and Supt. White--(they had been "detained" they said), all the seats were full; the only vacant seats in the house were three or four on the rostrum, reserved for and labeled "Ex-Presidents." Marching up to these, the three distinguished doctors seated themselves, unconscious of the sensation their presence had created, till the band struck up "Hail to the Chief,"* and three cheers were given (in Spanish) for the "distinguished ex-Presidents." Bennett says Dr. —— got up and bowed, and wanted to make a speech, but Dr. —— caught him by the coat tail and pulled him back (tore his coat). At the theatre these same gentlemen were shown to the box reserved for celebrities, and a "special piece" was played *to* them and in their honor. All three are now suffering from hypertrophy of the cerebellum, and will not speak to less than a Major-General.

---

**Attention** is called to changes in the Faculty of the Sewanee Medical College. This school is admirably located for close scientific study and laboratory work,—such branches as have been too much neglected in the bustle and hurry of clinical teaching in the large cities, and without a knowledge of which no man can attain to the dignity of a scientific physician. It is a summer school, and amid its beautiful parks and mountain scenery,—in classic walls, and a climate unsurpassed for purity and salubrity one may devote himself to study without fatigue or enervation, undisturbed by the thousand distracting influences which disturb the student in a hot dusty city. Sewanee is an ideal place for summer residence. Moreover, living is good in the mountains of Tennessee, and board inexpensive. There "appetite waits on digestion," and having something good to eat, one enjoys it, undisturbed by hints of indigestion and migraine. The session begins March 15. Write to Prof. H. W. Blanc, the Dean, for catalogue.

---

**The Afro-American in Medicine**, or "Colored Physician," is the name of a book, the prospectus of which the JOURNAL has received, to appear shortly. It is to be written by Dr. M. A. Majors, a colored physician of Waco, Texas. In this book, the prospectus says, are "depicted the lives and successes of the Afro-American in the devious paths of medical science, embracing Practice, Surgery, Dentistry and Pharmacy." It is to be illustrated by portraits of "all the leading colored physicians of the U. S." The author says:

"Twenty-seven years of freedom and education has not only

---

*There is difference on this point. Some say the band played "Annie Laurie,"—some say, "Lo the Conquering Hero Comes,"—others say, "Where Did You Get That Hat."—Ed.

made the negro a man, but a scholar in every avenue of the world's progress, there to meet on one common level the master minds of earth.

" We are not now so likely as a few years ago, to be measured by the number of pounds we might raise from the earth, or by the number of rails we might split in a day, but measured by that greater power (knowledge) which is required to move the world."

This is a commendable enterprise, and the JOURNAL wishes Dr. Majors abundant success."

---

The Progress of Pharmacy.—*Therapeusis of Piperazin.*—Accepting the very clear and complete clinical researches of Biesenthal, Schweninger, Ebstein, Vogt, Gautrelot, Heubach, Bardet and other well-known physicians, general practitioners have made many interesting tests of Piperazin and have arrived at some very satisfactory conclusions concerning its value. Its chief therapeutic indication is the uric acid diathesis, or the dyscrasia resulting from that condition. It is, unquestionably, the most energetic solvent of uric acid, and uratic concrementa which may be employed within the human organism without producing toxic effects. With uric acid it forms a neutral, soluble combination, while, at the same time, it dissolves the various albuminoids and their homologues. Prescribed in combination with Phenacetine it has very marked influence upon the gouty condition and promotes the absorption of undesirable exudates. The value of Piperazin in both acute and chronic gout, appears to be very decided. Schweninger reports success in 92% of his cases, and states that he could get no such results with any other remedy. Bissenthal also administered Piperazin in gout, in renal colic, and in urinary hemorrhages with perfect success. He gave it in carbonic acid water 1 to 500. The ordinary daily dose of Piperazin is fifteen grams. Some clinicians begin with three grains per diem, or 1 grain doses t. i. d.

A great drawback in the employment of Piperazin has arisen from the fact, that while in many cases, its use must be continued for a certain length of time in order to obtain its best effects, the cost of the medicament has been so high as to practically preclude its general use. It is gratifying to learn that through the enterprise of the Farbenfabriken, vorm, Friedr. Bayer & Co. (whose laboratories are at Elberfeld), a new process for the preparation of Piperazin has been discovered, and through the use of that method, the cost of this valuable new remedy has been reduced to about one-half of its former price.

Descriptive pamphlets of this product may be obtained from W. H. Schieffelin & Co., New York, who are the agents for this well-known laboratory. Other products of the Farbenfabriken, such as Phenacetine, Sulfonal, Europhen, and the later product, Salophen, are now being frequently employed in general practice.

*Sulfonal as a Substitute for Bromide of Potassium.*—Dr. Hinsdale of Philadelphia has reported a number of cases of the successful use of Sulfonal in cases usually treated by the bromides, and, especially, the brome of potassium. His conclusions are as follows: "The best results from Sulfonal are in those cases where the bromides cause so much skin trouble or mental disorder that their quantity must be lessened or altogether suspended; then Sulfonal becomes valuable." In a discussion upon Dr. Hinsdale's report, Dr. Dercum said: "Sulfonal has, in my experience, a decided value in diminishing the number of epileptic attacks. In a number of instances I have succeeded in markedly diminishing both the violence and the frequency of the seizures by very moderate doses of the drug, and its chief value seems to be that it can be administered for quite long periods in place of the bromides, thus enabling the patient to rally from the depressing effects of the latter."

---

### Association of Southern Medical Colleges.

Rules and Regulations* for the organization and maintenance of the Southern Medical College Association:

This Association shall be composed of delegates from Southern medical colleges, whose faculties have signified a desire to become members thereof, signed these rules of organization, and paid the membership fee of $5.00.

The objects of the Association are to cultivate closer and more intimate relations between medical colleges, and to elevate the standard of medical education by requiring a more thorough preliminary training, and an increased length of time of medical study.

---

*Adopted at the meeting of Southern Medical Colleges at Louisville, Nov. 16, 17, 1892.

The Association shall be composed of one or more delegates from each medical college belonging thereto, who shall be e[illegible]ted annually by their respective faculties. Each college shall be entitled to one vote in the transactions of the Association.

The officers shall consist of a President, Vice-President, Secretary and Treasurer, who shall be elected annually, just before the adjournment of the annual meetings, and shall perform the respective duties pertaining to these offices in similar organizations.

The meetings of the Association shall be held at the same time and place of the meetings of the Southern Surgical and Gynecological Association, unless otherwise determined by the Association.

### REQUIREMENTS FOR MATRICULATION.

Every student applying for matriculation must possess the following qualifications:

He must hold a certificate as the pupil of some known reputable physician, showing his moral character, and general fitness to enter upon the study of medicine.

He must possess a diploma of graduation from some literary or scientific institution of learning, or certificate from some legally constituted high school, general superintendent of State education or superintendent of some country board of public education, attesting the fact that he is possessed of at least the educational attainments required of second grade teachers of public schools. Provided, however, that, if a student so applying is unable to furnish the above and foregoing evidence of literary qualifications, he may be permitted to matriculate and receive instructions as other students, and qualify himself in the required literary departments, and stand his examination as above specified, prior to offering himself for a second course of lectures.

The foregoing diploma or certificate of educational qualifications, attested by the dean of the medical college attended, together with a set of tickets showing that the holder has attended one full course of medical lectures, shall be essential to attendance upon a second course of lectures in any college belonging to this Association.

*Branches of Medical Science to be Included in Course of Instructions*—Anatomy, physiology, chemistry, materia medica and therapeutics, theory and practice of medicine, pathology, sur-

